# The Alpha-Melanocyte-Stimulating Hormone Suppresses TLR2-Mediated Functional Responses through IRAK-M in Normal Human Keratinocytes

**DOI:** 10.1371/journal.pone.0136887

**Published:** 2015-08-26

**Authors:** Sunhyo Ryu, Andrew Johnson, Yoonkyung Park, Beomjoon Kim, David Norris, Cheryl A. Armstrong, Peter I. Song

**Affiliations:** 1 Department of Dermatology, University of Colorado Denver Anschutz Medical Campus, Aurora, Colorado, United States of America; 2 Department of Biotechnology, Chosun University School of Medicine, Gwangju, South Korea; 3 Department of Dermatology, University of Arkansas for Medical Sciences, Little Rock, Arizona, United States of America; 4 Department of Dermatology, Chung-Ang University School of Medicine, Seoul, South Korea; 5 Division of Dermatology, Denver Health Medical Center, Denver, Colorado, United States of America; Boston University School of Medicine, UNITED STATES

## Abstract

Alpha-melanocyte stimulating hormone (α-MSH) is a highly conserved 13-aa neuropeptide derived from pro-opiomelanocortin by post-translational processing, which has been reported to exhibit potent anti-inflammatory activity and a wide range of immunosuppressive activities in the skin. However, the regulatory effect of α-MSH is not completely clear in cutaneous innate immunity. In this study, we investigate the functional regulation of α-MSH in TLR2-mediated inflammatory responses in normal human keratinocytes (HKs). α-MSH pretreatment down-regulated the *Staphylococcus aureus* LTA-induced expression of both TLR2 and IL-8 as well as NF-κB nuclear translocation in HK cells. The inhibitory effect of α-MSH was blocked by agouti signaling protein (ASP), an α-MSH receptor-1 antagonist. To investigate the mechanism of this response in more detail, siRNA of IRAK-M, a negative regulator of TLR signaling, was utilized in these studies. The α-MSH suppressive effect on IL-8 production and NF-κB transactivation was inhibited by IRAK-M siRNA transfection in HK cells. These results indicate that α-MSH is capable of suppressing keratinocyte TLR2-mediated inflammatory responses induced by *S*. *aureus*-LTA, thus demonstrating another novel immunomodulatory activity of α-MSH in normal human keratinocytes.

## Introduction

Alpha-melanocyte-simulating hormone (α-MSH) is an endogenous tridecapeptide neurohormone derived from proopiomelanocortin (POMC), which participates in modulating cutaneous inflammatory and immune responses in normal human keratinocytes, langerhans cells, melanocytes and dermal fibroblasts [1, 2]. α-MSH exerts multiple biological effects on regulating cell proliferation, melanogenesis, immunomodulation, and cytoprotection in the skin through melanocortin 1 receptor (MC1R), a specific G-coupled protein receptor [3]. It was previously reported that α-MSH prevents TNFα-induced NF-κB transactivation through MC1R in human dermal fibroblast cells [4]. MC1R activation increases intracellular cAMP by stimulating adenylyl cyclase, which can be blocked by agouti signaling protein (ASP), a MC1R antagonist [5]. α-MSH has been reported to be a potent inhibitor of acute and chronic inflammation in a number of tissues [6], and it also inhibits the functional expression of immunoregulatory and pro-inflammatory cytokines such as IL-2, IFN-γ, TNFα, IL-6, and ICAM-1 [7]. As an antimicrobial peptide, α-MSH has also been reported to inhibit *Staphylococcus aureus* colony formation, and to reduce not only *Candida albicans* viability but also its germ tube formation [8]. Topical, subcutaneous, or intravenous administration of α-MSH inhibits contact hypersensitivity (CHS) via up-regulation of IL-10 [9].

Toll-like receptors (TLRs) participate in both inflammatory responses and innate host defense, and TLRs 2, 3, 4, 5 and 9 are functionally expressed in normal human keratinocytes [10–13]. TLR2 is the primary receptor involved in skin inflammatory responses to gram-positive bacteria such as *Staphylococcus aureus* (*S*. *aureus*). TLR2 is increased in keratinocytes on the skin lesions of acne, psoriasis, leprosy, and mycosis fungoides [14–17]. *S*. *aureus* is one of the commensal bacteria on the epithelial surfaces of human skin carried by 20–30% of the general human population [18–20]. *S*. *aureus* plays a role in a variety of dermatological diseases such as impetigo, cellulitis folliculitis, abscesses, atopic dermatitis, and psoriasis, when the epithelial barrier is breached. *S*. *aureus*-derived lipoteichoic acid (LTA) plays a major role in initiation and progression of infection by this organism as a key TLR2 ligand [18–21].

Although α-MSH has been reported to suppress NF-κB activation induced by various inflammatory agents, the mechanism of α-MSH-mediated functional regulation in *S*. *aureus* LTA-induced inflammatory responses is not completely clear in normal human keratinocytes. Thus, we investigate in this study the potential regulatory role of α-MSH in HK TLR2-mediated functional responses induced by *S*. *aureus* LTA.

## Materials and Methods

### Cell culture

Normal human keratinocytes (HKs) from foreskin were purchased from PromoCell (Heidelberg, Germany) and cultured in supplemented keratinocyte growth medium at 37°C in 5% CO_2_ as described in detail previously [22]. Cultured HK cells were propagated to at least 70% confluence, and, if required, were treated with *Staphylococcus aureus*-derived LTA (10 μg/ml; Sigma-Aldrich, St. Louis, MO), which were pre-incubated with α-MSH (10^−7^ M; Sigma-Aldrich, St. Louis, MO) for 2 hours in the presence or absence of agouti signaling protein (ASP, 10^−7^ M; Phoenix Pharmaceutical Inc., Burlingame, CA) as indicated in the results.

### Determination of the expression of TLR2 and IL-8 mRNA by real-time RT-PCR, and IL-8 protein by ELISA

The expression of TLR2 and IL-8 mRNA was measured by real-time RT-PCR 3 hours after treatment with LTA with or without pre-incubation with 10^−7^ M α-MSH for 2 hours in the presence or absence of ASP. Target gene mRNA expression was analyzed by real-time RT-PCR as described in the manufacturer’s protocol (ABI 7500 real-time PCR system using SYBR Green master mix; Applied Biosystems, Foster City, CA) as described in detail previously [22]. Oligonucleotide primers used to amplify human IL-8 and TLR2 cDNA were designed using the manufacturer's software (Primer Express 3.0; Applied Biosystems) based on published sequences [23, 24]. Quantification of target gene expression was normalized using an internal control gene, 18S rRNA [25]. The IL-8 primer sequences were 5'-GCAGTTTTGCCAAGGAGTGCT-3' (sense) and 5'-TTTCTGTGTTGGCGCAGTGTG-3' (antisense). The TLR2 primer sequences were 5’-TGTCTTGTGACCGCAATGGT-3’ (sense) and 5’-TGTTGGACAGGTCAAGGCTTT-3’ (antisense). The 18S rRNA primer sequences were 5'-CGGCTACATCCAAGGAA-3' (sense) and 5'-GCTGGAATTACCGCGGCT-3' (antisense).

To quantitatively measure IL-8 protein, HK cell supernatants were tested by ELISA using the Quantikine human IL-8 immunoassay kit (R&D Systems, Minneapolis, MN) according to the manufacturer’s instructions as previously described [13]. Cultured HK cell supernatants were collected 24 hours after LTA treatment. PBS and 500 ng/ml PMA served as negative and positive controls, respectively. All experiments were performed in triplicate.

### Determination of TLR2, IRAK-M and NF-κB protein expression in HK cells by Western blot analysis

Cultured HK cells were pre-incubated with or without α-MSH 10^−7^ M for 2 hours followed by treatment with LTA 10 μg/ml for 30 min, 60 min, 90 min and 24 hours. Cell lysis, and blotting were as described in detail previously [26, 27]. The membrane was blotted with a specific antibody to anti-human TLR2, anti-human IRAK-M, anti-human histone H3 antibodies (Cell Signaling, Danvers, MA), anti-human β-actin (Calbiochem, Gibbstown, NJ), and anti-human NF-κB antibody (rel A) (Rockland, Gilbertsville, PA).

### Immunofluorescence analysis to detect cellular localization of NF-κB, and IRAK-M

Cultured HK cells in 8-well chamber slides (10^4^ cells per well; Nalgene, Rochester, NY) were pre-incubated with or without α-MSH 10^−7^ M for 2 hours followed by treatment with LTA 10 μg/ml for 30 minutes. Immunofluorescence analysis to determine cellular localization of NF-κB localization and cellular localization was performed as previously described [13, 28]. Briefly, HK cells were incubated with a specific antibody to anti-human NF-κBp65 (Rockland, Gilbertsville, PA), and anti-human IRAK-M (cell signaling, Danvers, MA), and subsequently incubated for 1 hour at room temperature in the dark with FITC-conjugated affinity-purified goat anti-rabbit IgG (H+L; Jackson ImmunoResearch Laboratories, INC., West Grove, GA), which was diluted 1:300. The cells were visualized with the Zeiss fluorescent microscopic camera (Zeiss MicroImaging, Inc., Thornwood, NY).

### Statistical analysis

Results are expressed as mean±SD. For statistical analysis, ANOVA with probabilities were performed for both the overall significance (*P*) and the pair-wise comparison, indicated by asterisks. *P*<0.05 was considered to be significant.

## Results

### α-MSH suppressed LTA-induced expression of HK TLR2 and IL-8 through MCR1

**Fig 1:**
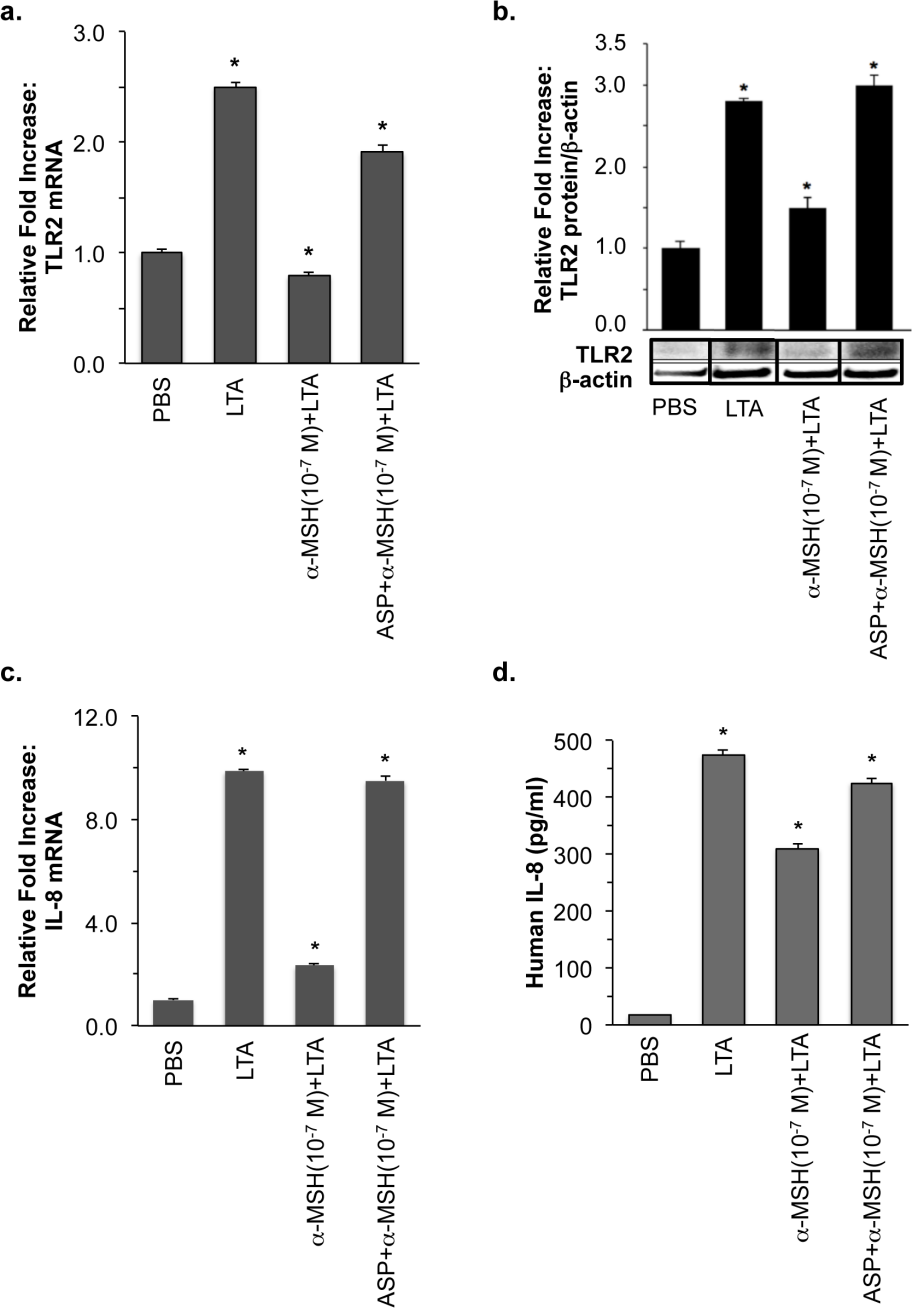
Inhibitory effect of α-MSH through MC1R on LTA-induced expression of HK TLR2 and IL-8. HK cells were treated with LTA, which were pre-incubated with α-MSH for 2 hours in the presence or absence of ASP as described in “Materials and Methods”. (**a**) The expression of HK TLR2 mRNA was measured by real-time RT-PCR 3 hours after *S*. *aureus*-derived LTA treatment. (**b**) TLR2 protein expression was determined by Western blot analysis using specific anti-human TLR2 polyclonal antibody (1:1000 dilution) and anti-β-actin antibody (1:5000 dilution) 24 hours after LTA treatment. (**c**) The expression of HK IL-8 mRNA was measured by real-time RT-PCR 3 hours after *S*. *aureus*-derived LTA treatment. (**d**) LTA-induced HK IL-8 secretion was measured by ELISA. HK treated with PBS served as a negative control. The relative intensity of expression was normalized using the expression of 18S rRNA for mRNA and β-actin for protein as internal controls. All values are expressed as mean ± SD. Statistically significant differences in the expression of HK TLR2 were determined by ANOVA with probabilities shown for both the overall significance and the pairwise comparison (*P<0.001).

To analyze α-MSH effect on TLR2 expression induced by a major staphylococcal cell wall component, HK cells were treated with 10 μg/ml *S*. *aureus*-derived LTA in the presence or absence of 10^−7^ M α-MSH. As shown in Fig 1, more than 2.5 fold-increased expression of TLR2 mRNA and its proteins, which was induced by LTA for 3 and 24 hours, respectively, was efficiently blocked by α-MSH treatment.

Since it was previously reported that α-MSH pre-incubation significantly reduced LTA-induced IL-8 expression in HaCaT transformed keratinocytes [29], we examined α-MSH suppression of LTA-induced IL-8 expression in primary normal human keratinocytes. LTA-induced 10 fold-increased IL-8 mRNA was significantly inhibited up to 2 of the relative fold increase by α-MSH (Fig 1C). The increased amount of secreted HK IL-8 protein, which was induced by 24-hour LTA treatment, was also significantly suppressed from 500 to 300 pg/ml by α-MSH (Fig 1D). The α-MSH inhibitory effect was released by treatment with agouti signaling protein (ASP), a MC1R antagonist (Fig 1). These results demonstrate that the increased expression of HK TLR2 and IL-8 induced by *S*. *aureus*-derived LTA is specifically down-regulated by α-MSH.

### α-MSH inhibited LTA-induced NF-κB nuclear translocation in HK cells

**Fig 2:**
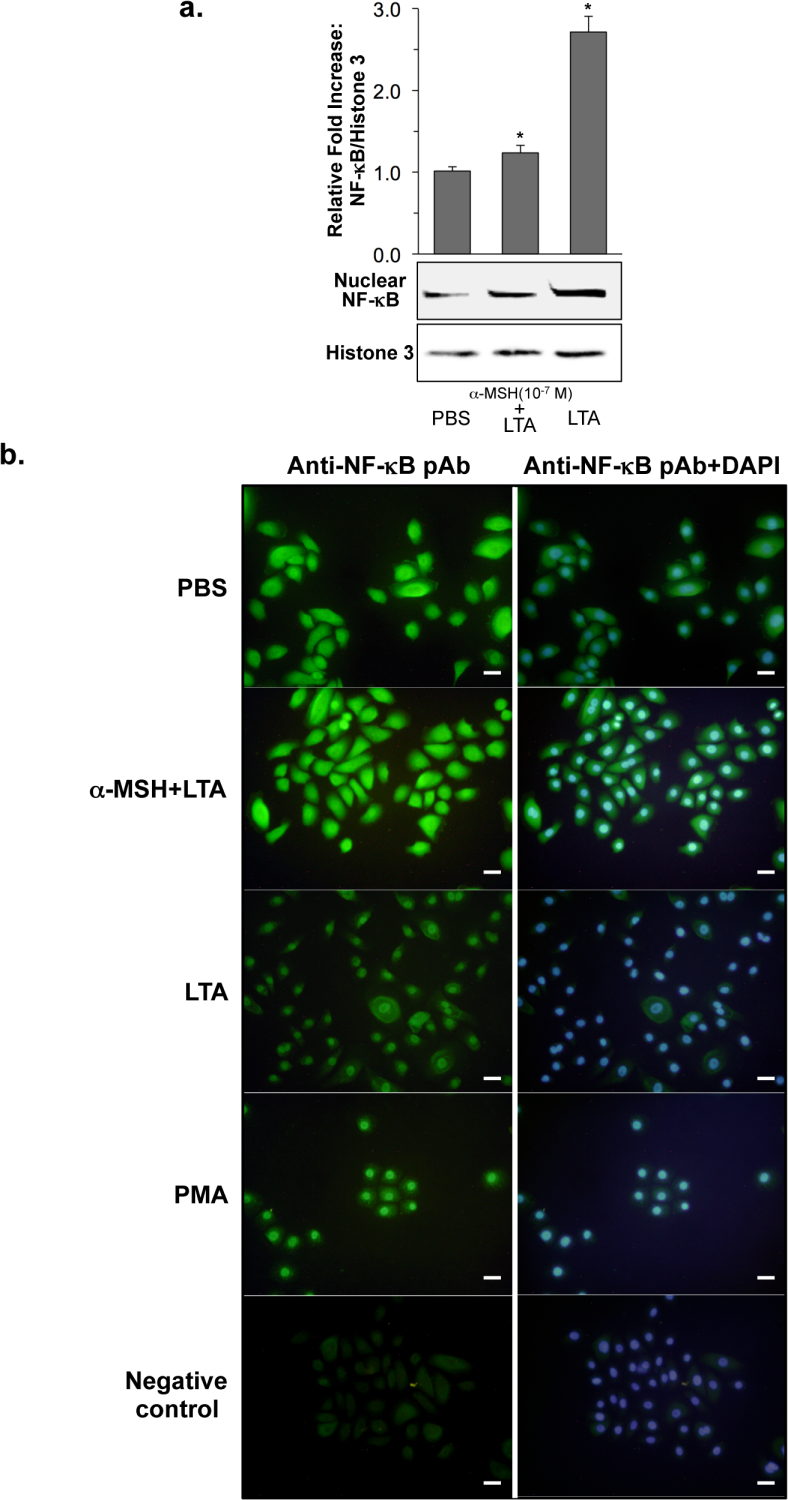
Inhibitory effect of α-MSH on LTA-induced NF-κB nuclear translocation. (**a**) Nuclear localization of NF-κB was determined by Western blot. Nuclear extracts of HK cells were prepared 1 hour after LTA treatment, which were pre-incubated with α-MSH for 2 hours, and were subjected to Western blot analysis using specific anti-NF-κB antibody (Rel A (1:2500 dilution) and anti-histone H3 antibody (1:1000 dilution). The relative intensity of NF-κB was normalized using histone H3 expression as an internal control. (**b**) Cellular localization of NF-κB was determined by immunofluorescent staining of NF-κB as described in “Materials and Methods”. NF-κB was detected using specific anti- NF-κBp65 polyclonal antibodies (1:200 dilution) for its intracellular localization (green), which was compared with Hoechst-stained nuclei (blue). HK treated with PBS and 50 ng/ml PMA served as a negative and a positive control, respectively. HK incubated with FITC-conjugated anti-rabbit IgG(H+L) served as a technical negative control. Bars = 20 μm.

To determine α-MSH regulation of NF-κB transcriptional activation, which is closely associated with TLR2 signaling pathway [30], we quantified nuclear NF-κB by Western blot. The 2.5 fold-increased amount of nuclear NF-κB, which was induced by LTA, was significantly reduced to 1.2 fold by α-MSH treatment (Fig 2A). We next analyzed LTA-induced NF-κB nuclear translocation by immunofluorescent staining in the presence or absence of α-MSH. As shown in Fig 2B, LTA treatment increased HK NF-κB nuclear translocation. In contrast, α-MSH efficiently suppressed the LTA-induced NF-κB nuclear translocation. Phorbol 12-myristate 13-acetate (PMA), a stimulus of pro-inflammation through NF-κB transcription factor [30], served as a positive control in this study.

### IRAK-M expression was induced by α-MSH in HK cells

**Fig 3:**
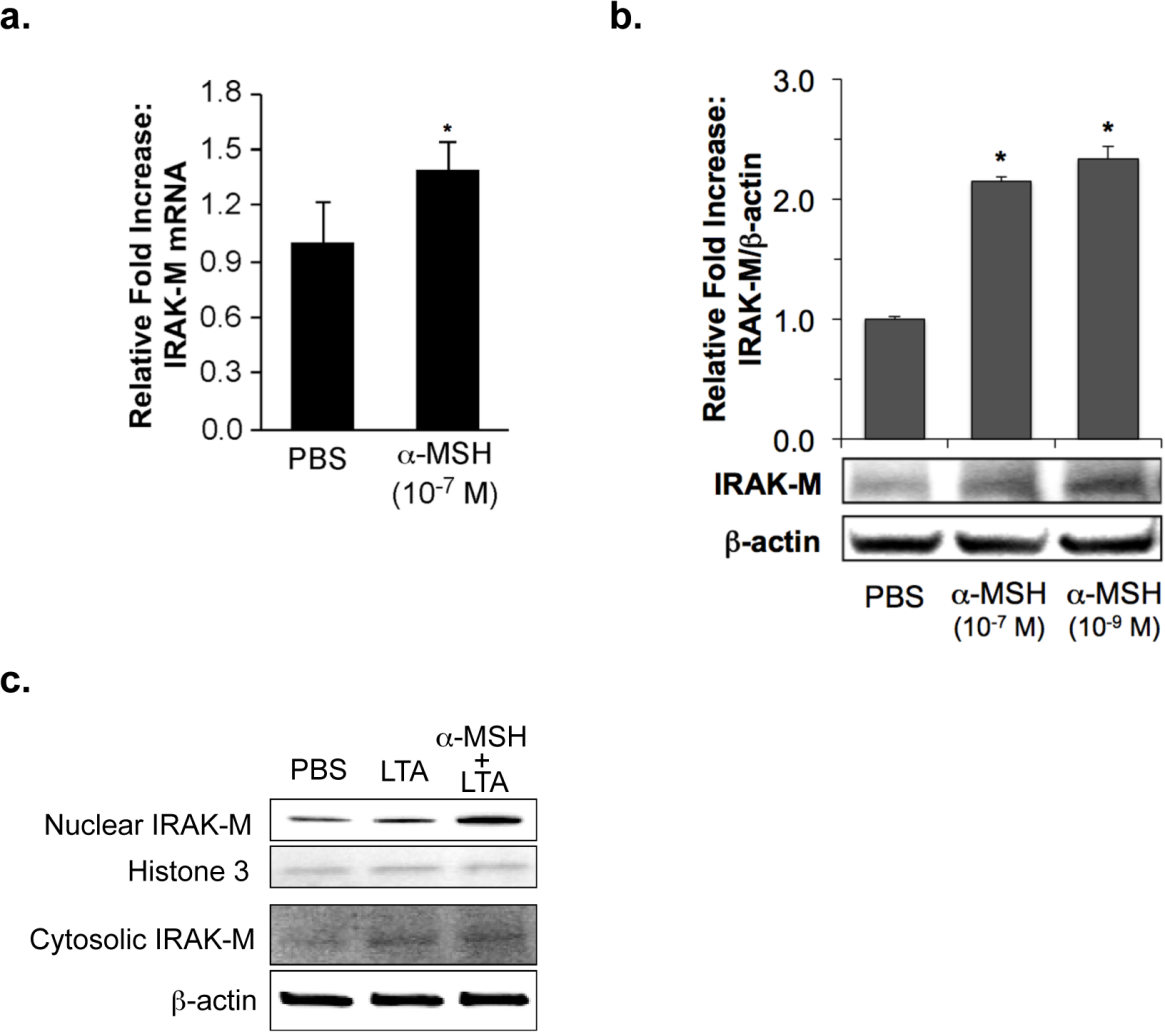
HK IRAK-M expression induced by α-MSH. The expression of IRAK-M mRNA (**a**) and its protein (**b**) induced by α-MSH was determined by real-time RT-PCR and Western blot, respectively, as described in “Materials and Methods”. PBS-treated HK cells served as the negative control. The relative intensity of expression was normalized using the expression of 18S rRNA for mRNA and β-actin for protein as internal controls. All values are expressed as mean ± SD. Statistically significant differences in the expression of HK IRAK-M were determined by ANOVA with probabilities shown for both the overall significance and the pairwise comparison (*P<0.001). (**c**) The HK cellular localization of IRAK-M was determined by Western blot using specific anti-IRAM-M antibody (1:1000 dilution) as described in “Materials and Methods”. The relative intensity of IRAK-M in cytoplasmic and nuclear extracts of HK cells was normalized using β-actin and histone H3 expression, respectively.

IRAK-M is a potential negative regulator of TLR signaling [27], and α-MSH was previously reported to suppress LPS-stimulated TLR4 activation through IRAK-M in macrophages [31]. Since α-MSH decreased HK IL-8 expression, which was associated with LTA-TLR2 signaling in this study, we tested a regulatory role of IRAK-M in the α-MSH suppression in LTA-treated HK cells. When we examined HK IRAK-M expression in the presence or absence of α-MSH, the expression of IRAK-M mRNA and its proteins was significantly induced by 10^−7^ M α-MSH (Fig 3A and 3B). The amount of HK nuclear IRAK-M was increased by α-MSH treatment in LTA-stimulated HK cells. In contrast, cytosolic IRAK-M was not clearly changed in the presence or absence of α-MSH (Fig 3C).

### The α-MSH suppressive effect on IL-8 production was inhibited by transfection of HK cells with IRAK-M siRNA

**Fig 4:**
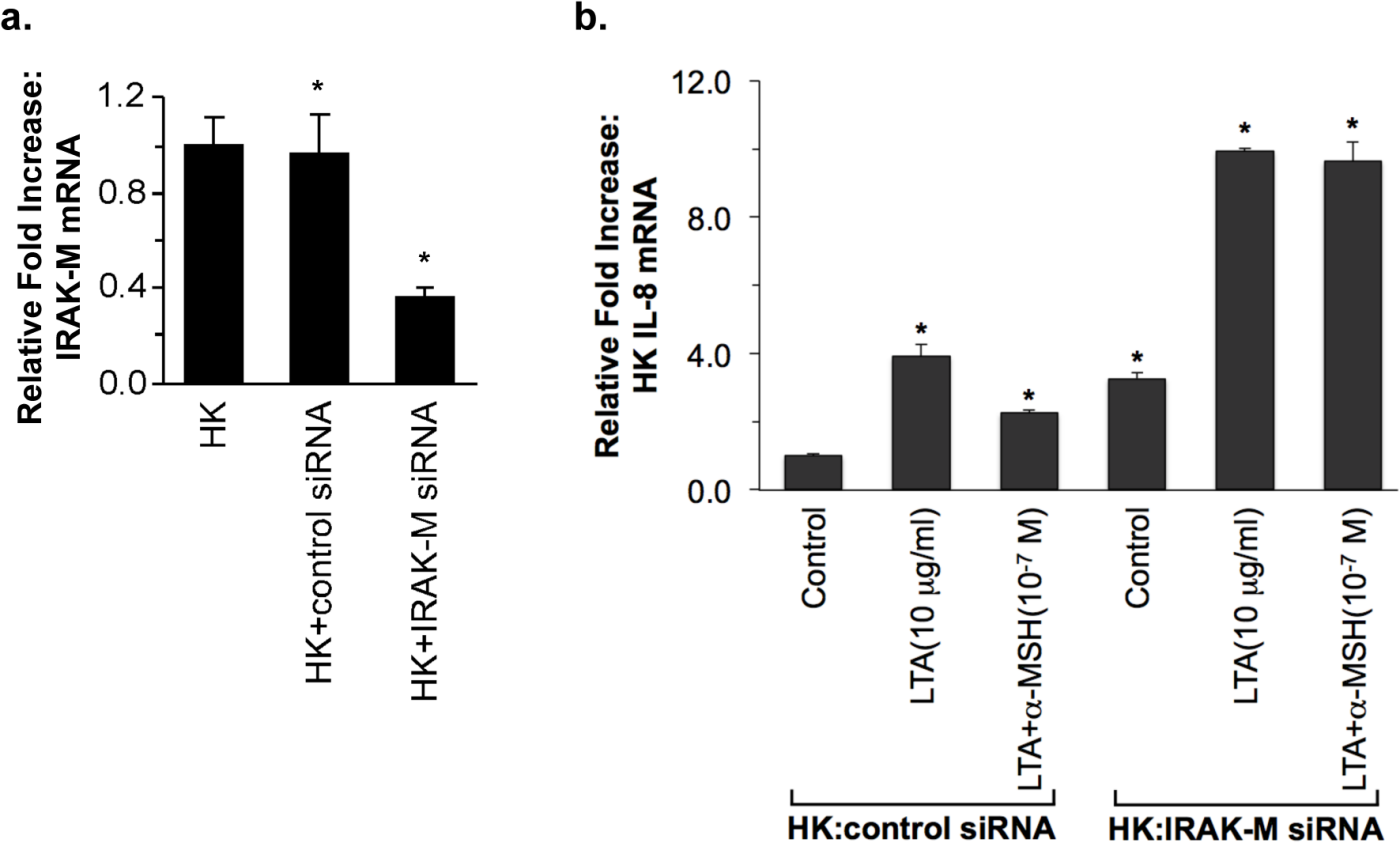
Inhibitory effect of IRAK-M siRNA on α-MSH-suppressed HK IL-8 expression. HK cells were transiently transfected with IRAK-M siRNA or control siRNA prior to LTA treatment. The mRNA expression of IRAK-M (**a**) and IL-8 (**b**) was determined by real-time RT-PCR 3 hours after LTA treatment in the presence or absence of α-MSH. The relative intensity of expression was normalized using the expression of 18S rRNA. All values are expressed as mean ± SD. Statistically significant differences in the expression of HK IRAK-M and IL-8 were determined by ANOVA with probabilities shown for both the overall significance and the pairwise comparison (*P<0.001).

We next examined a regulatory effect of IRAK-M on IL-8 production, which is one of the LTA-induced inflammatory signaling responses [27, 30]. When keratinocytes were transiently transfected with IRAK-M siRNA, the mRNA expression of HK IRAK-M was significantly blocked in comparison with its level in control siRNA transfected HK cells (Fig 4A). As shown in Fig 4B, the expression of IL-8 mRNA was significantly increased by LTA treatment in IRAK-M siRNA- and control siRNA transfected HK cells. The level of IL-8 increase was much higher in IRAK-M siRNA transfected HK cells than that of control siRNA-transfected cells. The LTA-induced IL-8 mRNA increase in control siRNA-transfected HK cells was significantly inhibited by 10^−7^ M α-MSH. In contrast, the α-MSH suppression was not observed in IRAK-M siRNA transfected HK cells (Fig 4B).

### The α-MSH suppressive effect on NF-κB nuclear translocation was inhibited by transfection of HK cells with IRAK-M siRNA

**Fig 5:**
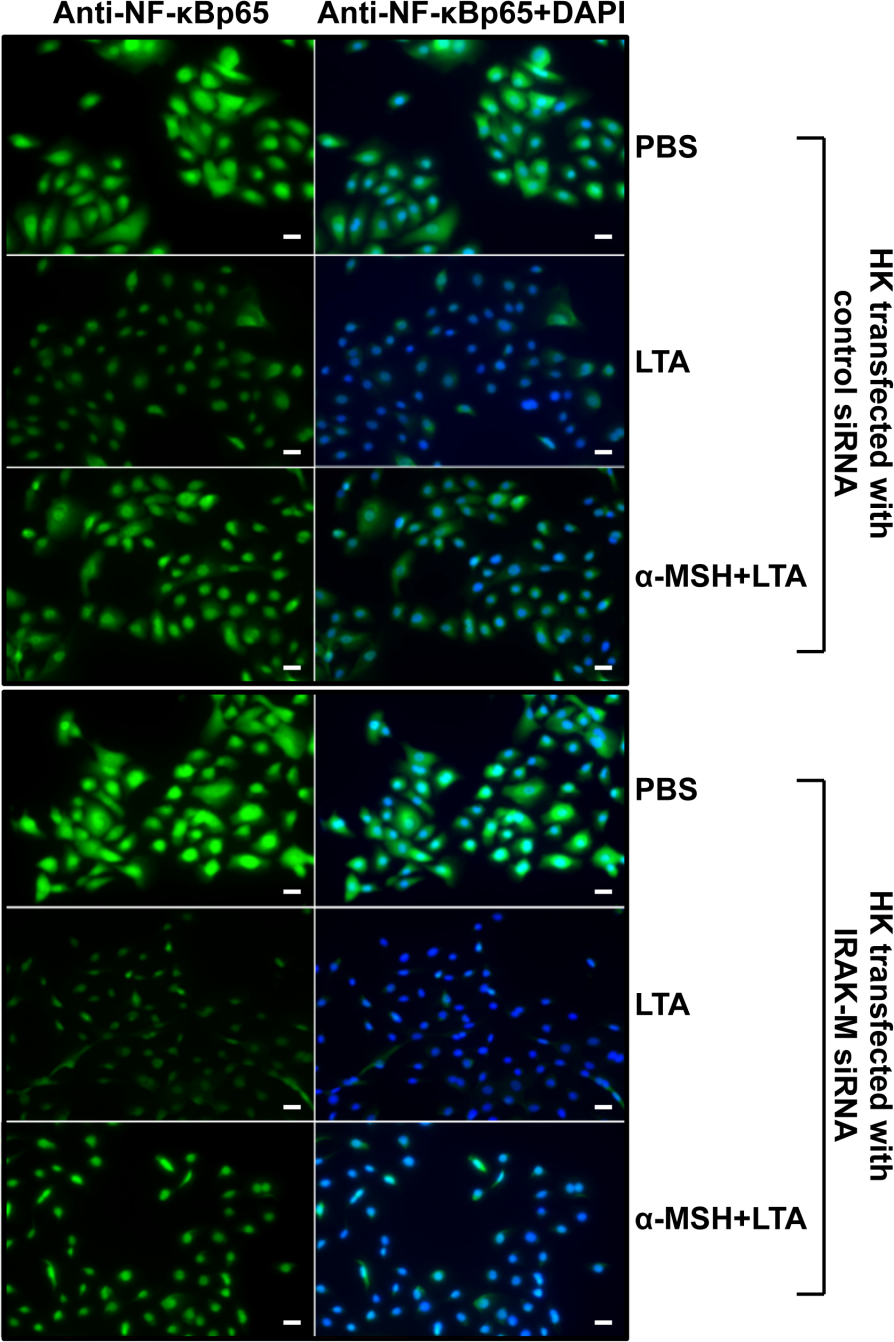
Inhibitory effect of IRAK-M siRNA on α-MSH-suppressed HK NF-κB nuclear translocation. After HK cells were transiently transfected with IRAK-M siRNA or control siRNA prior to LTA treatment, NF-κB cellular localization was determined by immunofluorescent staining using a specific anti-human NF-κBp65 antibody 30 minutes after LTA treatment in the presence or absence of α-MSH. The left column is the FITC fluorescent signal indicative of NF-κB, the center column is the DAPI nuclear stain, and the right column is the merged image demonstrating nuclear or cytoplasmic of NF-κB localization. PBS-treated HK cells served as the negative control. Bars = 20 μm.

In order to determine a more detailed regulatory mechanism of α-MSH in transcriptional activation of transiently transfected HK cells with IRAK-M siRNA, we analyzed NF-κB cellular localization of the keratinocytes by immunofluorescent staining using specific NF-κBp65 antibodies 30 minutes after LTA treatment in the presence or absence of α-MSH. As shown in Fig 5, the ratio of nuclear localized NF-κB was significantly increased by LTA in both control siRNA- and IRAK-M siRNA-transfected HK cells in the absence of α-MSH compared to that of PBS-treated control HK cells. In control siRNA-transfected HK cells, α-MSH efficiently suppressed the LTA-induced NF-κB nuclear translocation (Fig 5). However, the α-MSH suppressive level of LTA-induced NF-κB nuclear translocation was not clearly observed in IRAK-M siRNA-transfected keratinocytes. As shown in “S3 Fig”, the amount of nuclear localized NF-κB in IRAK-M siRNA-transfected HK cells, which were treated with α-MSH+LTA, is very similar with that of LTA-induced HK cells. These data indicate that NF-κB nuclear translocation may not be completely inhibited during the LTA-induced transcriptional activation in IRAK-M siRNA transfected HK cells. This is consistent with the nuclear NF-κB immunofluorescent staining results with anti-NF-κB polyclonal antibodies in Fig 5.

Taken together, these results indicate that α-MSH suppression of LTA-induced NF-κB transactivation is specifically associated with IRAK-M, which has a negative regulatory function in LTA-induced inflammatory signaling through TLR2.

## Discussion

The activation of TLR signaling pathways induced by specific pathogens ultimately results in NF-κB transactivation, followed by secreting inflammatory cytokines such as IL-1, IL-6, TNFα and IL-8, by which innate inflammatory immune responses are initiated. NF-κB signaling activation associated with TLRs is thought to be a pivotal link between the innate and adaptive immune systems since TLRs not only provoke the innate immune response and enhance adaptive immunity against pathogens, but also are involved in the pathogenesis of autoimmune and chronic inflammation [32]. It was previously reported that the treatment of LPS-stimulated macrophages with α-MSH inhibits the NF-κB nuclear translocation and p38 activation by blocking TLR4 signaling using the intracellular TLR-inhibitor IRAK-M [31, 33, 34]. Although human IRAK-M expression is restricted to monocytes and macrophages [26, 35], however, there is no known information about the regulatory effect of α-MSH on TLR2-mediated inflammatory responses in *S*. *aureus* LTA-induced keratinocytes. In this study, we specifically investigate the negative regulation of α-MSH in *S*. *aureus*-LTA induced TLR2-signaling activation in primary normal human keratinocytes.

TLR2 in association with TLR1 or TLR6 recognizes the indicated ligands such as lipoteichoic acid (LTA) and peptidoglycan [36]. Staphylococcal LTA-activated TLR2 recruits TIRAP, a Toll-interleukin 1 receptor (TIR) domain-containing adaptor protein, which links the TLR to MyD88. MyD88 in turn promotes an association with IRAK (IL-1 receptor- associated kinase) family members such as IRAK4, IRAK1, and IRAK2. IRAK4 is activated first, followed by sequential activation of IRAK1 and IRAK2 [37]. IRAK4 is known to be essential for TLR–IL-1R-mediated cellular responses as a serine-threonine kinase to eventually induce TLR-associated cytokine production [31, 38]. The activated IRAK family proteins associate with TRAF6 (TNF receptor-associated factor 6), and TRAF6 activates TAK1, which in turn activates the IKK complex composed of NEMO, IKKα and IKKβ. The activated IKK complex phosphorylates IκBα, which is then ubiquitinated and degraded by the proteasome, while NF-κB subunits p50 and p65 translocate to the nucleus. TAK1 also activates the MAPK signaling pathway. The activated NF-κB and MAPK in the nucleus initiate the transcription of inflammatory cytokine genes such as IL-8 [39]. As a chemotactic factor to recruit neutrophils at the site of inflammation, IL-8 is usually thought to be an activation marker of TLRs-associated inflammatory responses [40].

Donnarumma *et al*. previously reported that α-MSH reduces *S*. *aureus* internalization, and down-regulates HSP70, integrins, and the expression of ICAM-1 and pro-inflammatory cytokines such as IL-8 and TNFα in HaCaT keratinocyte cell line [29]. However, little is known about a specific mechanism of the α-MSH down-regulation in *S*. *aureus* LTA-stimulated primary human keratinocytes. Our results demonstrate for the first time that the increased expression of TLR2 and IL-8 mRNAs and their proteins, which is induced by *S*. *aureus*-derived LTA through NF-κB transcriptional activation, is specifically down-regulated by α-MSH in primary normal human keratinocytes (Figs 1 to 2).

The IRAK family consists of two active kinases, IRAK and IRAK-4, and two inactive kinases, IRAK-2 and IRAK-M. IRAK-M expression is restricted to monocytes/macrophages, whereas other IRAKs are ubiquitous [27, 41]. IRAK-M prevented dissociation of IRAK and IRAK-4 from MyD88 and formation of IRAK-TRAF6 complexes. IRAK-M^(-/-)^ cells exhibited increased cytokine production upon TLR/IL-1 stimulation and bacterial challenge, and IRAK-M^(-/-)^ mice showed increased inflammatory responses to bacterial infection [27]. We demonstrate in this study that primary normal human keratinocytes constitutively express IRAK-M; moreover, the expression of IRAK-M mRNA and its protein was induced by α-MSH treatment (Fig 3A and 3B, S1 Fig and S2 Fig). Su *et al*. previously reported that IRAK-M is present in both the cytoplamic and nuclear fractions in resting THP-1 cells [42]. The authors showed that bacterial lipoprotein Pam3 CSK4 challenge caused significant reduction of nuclear IRAK-M levels, indicating that IRAK-M may undergo nuclear export upon challenge [42]. We indicated in this study that the amount of HK nuclear IRAK-M was increased by α-MSH in PBS- and LTA-stimulated HK cells; in contrast, cytosolic IRAK-M was not clearly changed in the presence or absence of α-MSH in PBS- and LTA-treated keratinocytes (Fig 3C). In IRAK-M siRNA transfected HK cells, which express significantly reduced IRAK-M (Fig 4A), the level of IL-8 mRNA expression was greatly increased by LTA treatment. While the LTA-induced IL-8 increase was significantly blocked by α-MSH in control siRNA transfected HK cells, the α-MSH suppression was not observed in IRAK-M siRNA transfected HK cells (Fig 4B). Moreover, α-MSH suppressive effect on NF-κB nuclear translocation was also inhibited by transfection of HK cells with IRAK-M siRNA (Fig 5 and S3 Fig), indicating that α-MSH suppression of LTA-induced IL-8 increase through NF-κB transactivation may be specifically associated with a negative functional regulation of IRAK-M in the HK TLR2 signaling pathway.

In conclusion our results indicate that α-MSH is capable of suppressing HK TLR2-mediated inflammatory responses induced by *S*. *aureus*-derived LTA, thus demonstrating another novel immunomodulatory activity of α-MSH in primary normal human keratinocytes.

## Supporting Information

S1 Fig The mRNA expression of IRAK-M in normal human keratinocytes. The mRNA expression of IRAK-M in HK cells was determined by RT-PCR using specific oligonucleotide primers, which were designed using the manufacturer's software (Primer Express 3.0; Applied Biosystems) based on published sequences [43]. The IRAK-M primer sequences were 5’-GTTGATGGCACATCCCACGTC-3’ (sense) and 5’-GTACAGGGCATAGACATGGC-3’ (antisense). The PCR products of IRAK-M (196 bp) were analyzed by 1.5% agarose gelelectrophoresis.
(TIF)

S2 Fig HK IRAK-M immunofluorescent staining. The HK cellular localization of IRAK-M was determined by immunofluorescent staining using specific anti-IRAK-M (1:50 dilution) as described in “Materials and Methods”. THP-1 cells served as the positive control, and HK cells treated with the secondary antibody alone served as the negative control.
(TIF)

S3 Fig Nuclear localization of NF-κB determined by Western blot. Nuclear extracts of IRAK-M siRNA-transfected HK cells were pre-incubated with/without α-MSH for 2 hours, prepared 1 hour after LTA treatment, and then subjected to Western blot analysis using specific anti-NF-κB antibody (Rel A (1:2500 dilution) and anti-histone H3 antibody (1:1000 dilution). The relative intensity of NF-κB was normalized using histone H3 expression as an internal control. The amount of nuclear localized NF-κB in IRAK-M siRNA-transfected HK cells, which were treated with α-MSH+LTA, is very similar with that of LTA-induced HK cells. These data indicate that NF-κB nuclear translocation may not be completely inhibited during the LTA-induced transcriptional activation in IRAK-M siRNA transfected HK cells. This is consistent with the nuclear NF-κB immunofluorescent staining results with anti-NF-κB polyclonal antibodies in Fig 5.
(TIF)
