# Supplementary figures and images for: The Alpha-Melanocyte-Stimulating Hormone Suppresses TLR2-Mediated Functional Responses through IRAK-M in Normal Human Keratinocytes
Source: PLoS One. 2015 Aug 26;10(8):e0136887. doi: 10.1371/journal.pone.0136887 (PMC4550463; doi:10.1371/journal.pone.0136887)

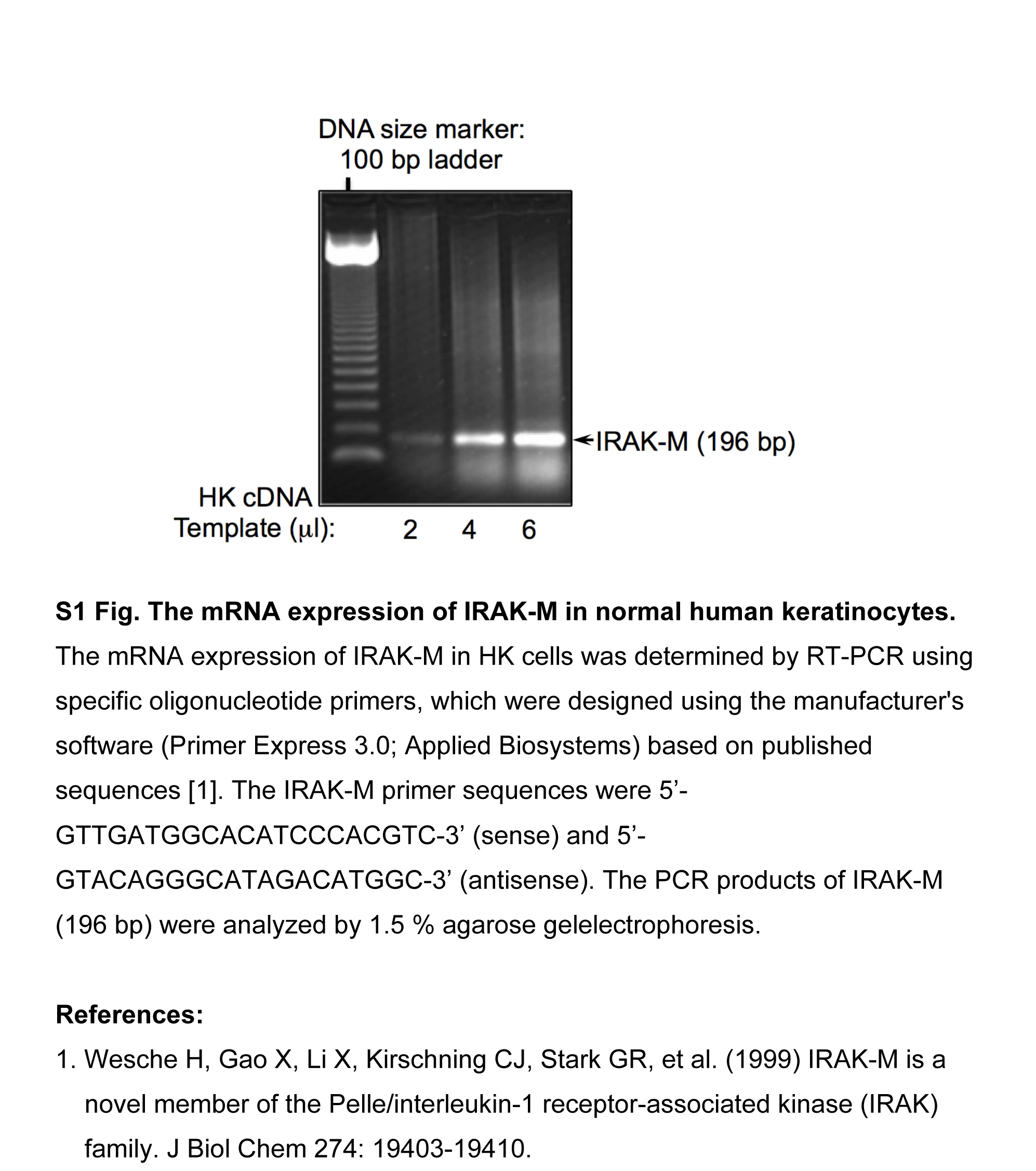

Supplement: S1 Fig — The mRNA expression of IRAK-M in HK cells was determined by RT-PCR using specific oligonucleotide primers, which were designed using the manufacturer's software (Primer Express 3.0; Applied Biosystems) based on published sequences [43]. The IRAK-M primer sequences were 5’-GTTGATGGCACATCCCACGTC-3’ (sense) and 5’-GTACAGGGCATAGACATGGC-3’ (antisense). The PCR products of IRAK-M (196 bp) were analyzed by 1.5% agarose gelelectrophoresis. (TIF) [file pone.0136887.s001.tif]

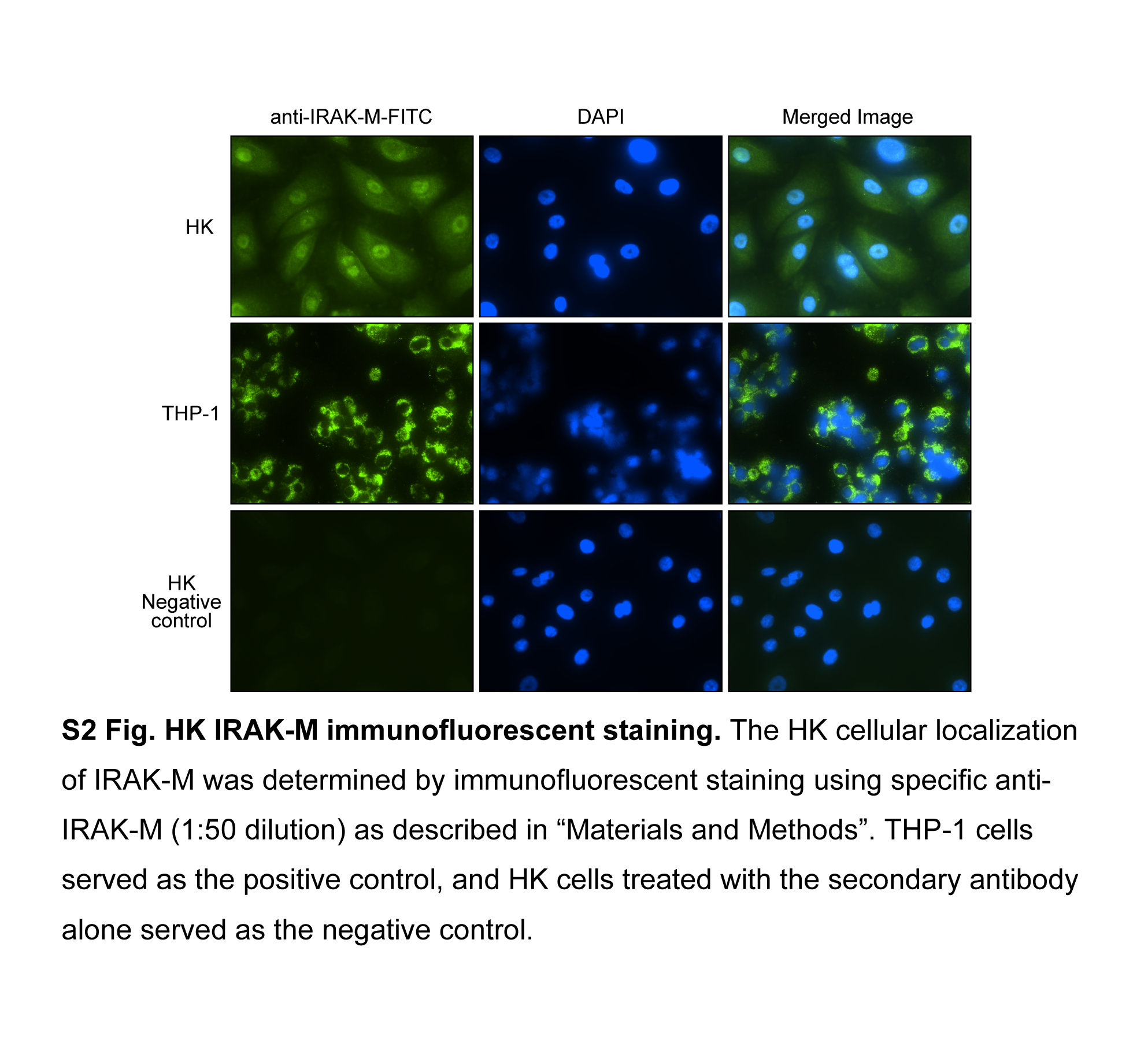

Supplement: S2 Fig — The HK cellular localization of IRAK-M was determined by immunofluorescent staining using specific anti-IRAK-M (1:50 dilution) as described in “Materials and Methods”. THP-1 cells served as the positive control, and HK cells treated with the secondary antibody alone served as the negative control. (TIF) [file pone.0136887.s002.tif]

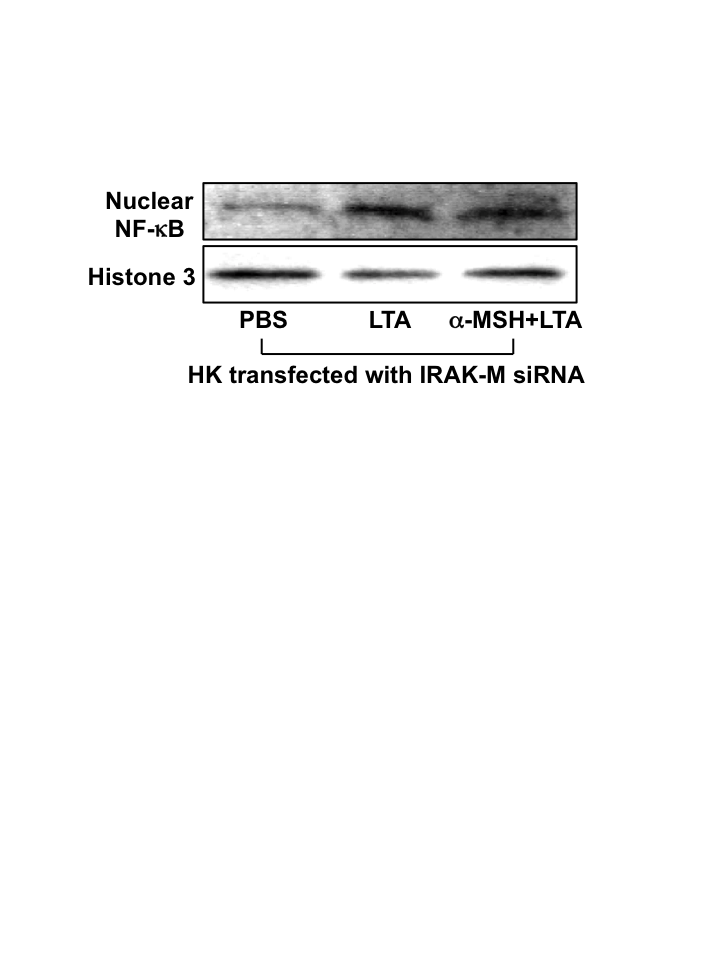

Supplement: S3 Fig — Nuclear extracts of IRAK-M siRNA-transfected HK cells were pre-incubated with/without α-MSH for 2 hours, prepared 1 hour after LTA treatment, and then subjected to Western blot analysis using specific anti-NF-κB antibody (Rel A (1:2500 dilution) and anti-histone H3 antibody (1:1000 dilution). The relative intensity of NF-κB was normalized using histone H3 expression as an internal control. The amount of nuclear localized NF-κB in IRAK-M siRNA-transfected HK cells, which were treated with α-MSH+LTA, is very similar with that of LTA-induced HK cells. These data indicate that NF-κB nuclear translocation may not be completely inhibited during the LTA-induced transcriptional activation in IRAK-M siRNA transfected HK cells. This is consistent with the nuclear NF-κB immunofluorescent staining results with anti-NF-κB polyclonal antibodies in Fig 5. (TIF) [file pone.0136887.s003.tif]
